# Laurentian Great Lakes Phytoplankton and Their Water Quality Characteristics, Including a Diatom-Based Model for Paleoreconstruction of Phosphorus

**DOI:** 10.1371/journal.pone.0104705

**Published:** 2014-08-08

**Authors:** Euan D. Reavie, Adam J. Heathcote, Victoria L. Shaw Chraïbi

**Affiliations:** 1 Center for Water and the Environment, Natural Resources Research Institute, University of Minnesota Duluth, Duluth, Minnesota, United States of America; 2 Groupe de Recherche Interuniversitaire en Limnologie, Département des Sciences Biologiques, Université du Québec à Montréal, Montréal, Québec, Canada; 3 Department of Earth and Atmospheric Sciences, University of Nebraska-Lincoln, Lincoln, Nebraska, United States of America; CNRS, France

## Abstract

Recent shifts in water quality and food web characteristics driven by anthropogenic impacts on the Laurentian Great Lakes warranted an examination of pelagic primary producers as tracers of environmental change. The distributions of the 263 common phytoplankton taxa were related to water quality variables to determine taxon-specific responses that may be useful in indicator models. A detailed checklist of taxa and their environmental optima are provided. Multivariate analyses indicated a strong relationship between total phosphorus (TP) and patterns in the diatom assemblages across the Great Lakes. Of the 118 common diatom taxa, 90 (76%) had a directional response along the TP gradient. We further evaluated a diatom-based transfer function for TP based on the weighted-average abundance of taxa, assuming unimodal distributions along the TP gradient. The r^2^ between observed and inferred TP in the training dataset was 0.79. Substantial spatial and environmental autocorrelation within the training set of samples justified the need for further model validation. A randomization procedure indicated that the actual transfer function consistently performed better than functions based on reshuffled environmental data. Further, TP was minimally confounded by other environmental variables, as indicated by the relatively large amount of unique variance in the diatoms explained by TP. We demonstrated the effectiveness of the transfer function by hindcasting TP concentrations using fossil diatom assemblages in a Lake Superior sediment core. Passive, multivariate analysis of the fossil samples against the training set indicated that phosphorus was a strong determinant of historical diatom assemblages, verifying that the transfer function was suited to reconstruct past TP in Lake Superior. Collectively, these results showed that phytoplankton coefficients for water quality can be robust indicators of Great Lakes pelagic condition. The diatom-based transfer function can be used in lake management when retrospective data are needed for tracking long-term degradation, remediation and trajectories.

## Introduction

Recent trends from monitoring data demonstrate rapid changes in the Laurentian Great Lakes system associated with anthropogenic drivers. Most notably, changes in the water quality [1], phytoplankton (unpublished data), zooplankton [2] and benthos [3] collectively confirm or suggest detriments associated with invasive species, nutrient imbalances and climate. While algal abundances have been inordinately low in some regions (e.g., Lake Huron; unpublished data), new eutrophication problems have also manifested as substantial algal blooms (e.g., [4]). Such quantitative assessments of environmental problems have been valuable to support lake management [5] because they provide information on the abundance of various organisms, traits that are critical to understanding and managing food webs. However, taxon-specific evaluations of pelagic indicators in the Great Lakes are lacking. In addition to these more intuitive evaluations of organism numbers and biomass, taxon-specific ecological data can provide additional tools to inform management [6].

Developing effective indicators of ecological condition requires that they be calibrated to identify their responses to important environmental stressors [7]. The main goals of calibration are to identify environmental characteristics of potential indicator taxa so that they may be subsequently used to infer condition. Assemblages of algae, which are physiologically subject to water quality, have the potential to provide time-integrated inferences of limnological conditions. Such bioindicators are particularly needed to monitor the impacts of human activities that are increasing nutrient supplies to water bodies, introducing non-native species, and changing climate. Great Lakes coastal algae, particularly diatoms, have been shown to provide a more temporally integrated assessment of water quality conditions than discrete water quality measurements [8], but similar indicators have not been developed for the pelagic ecosystem. Diatoms are particularly advantageous as indicators in paleolimnological studies because their diagnostic cell walls (frustules) remain archived in lake sedimentary records [9]. Hence, the fossil diatom assemblages may be used to reconstruct historical degradation and recovery and estimate future trajectories of condition.

The characteristics of the Great Lakes phytoplankton community as indicators of the prevailing eutrophication that was noted in the 1960s and 1970s was well described by Stoermer [10]. At that time little taxonomic treatment of the phytoplankton had been accomplished, so Stoermer advocated the indicator potential that would result from better taxonomic assessment of the flora and species autecology. Significant collections of phytoplankton have occurred since that time, such as the United States Environmental Protection Agency's (USEPA) biological monitoring program that has been active since 1983 [11], among other monitoring data (e.g., [12]). We aimed to provide the tools to develop species- and assemblage-based indicators from Great Lakes phytoplankton data.

Modern datasets (also known as training sets) provide the basis for developing indicator transfer functions by relating contemporary assemblages with their corresponding environmental measurements [13]. Algal assemblages, in particular, are proven robust indicators of stressors such as nutrients ([14],[15]), water clarity [16] and acidification [17], as well as a suite of other water quality problems in freshwater ecosystems [18]. Training sets using diatoms have been developed for lakes, rivers and the coastlines of the Great Lakes [8]. Algae are known to have definable optima along gradients of environmental conditions. Species tend to be taxonomically distinct and abundant, and they respond rapidly to changing conditions. Hence, by using training set data to calibrate the environmental characteristics of algae, researchers can use changes in community composition to classify and quantify long-term environmental changes that result from anthropogenic activities. This is particularly needed because few pelagic monitoring data were collected prior to the 1970s; existing data provide a spatially and temporally incomplete picture of environmental conditions in the lakes. Species-environmental coefficients should allow reconstructions of past conditions based on sedimentary assemblages.

A transfer function is derived by relating taxa assemblages (usually diatoms) in a training set of samples (in this case, pelagic samples collected throughout the Great Lakes) to an environmental variable of interest [19]. The transfer function consists of taxa coefficients (environmental optima and, optionally, tolerances) that can be used to infer quantitative information about the variable of interest based on the abundance of each taxon in a sample assemblage. These assemblages are usually characterized from recently accumulated surface sediments [20]; to our knowledge, a transfer function based on diatom phytoplankton samples has not been attempted.

For the current assessment, modeling approaches were applied to characterize the environmental coefficients of the Great Lakes phytoplankton taxa. A checklist of the common taxa and their ecological indicator values for a suite of water quality variables is presented. To illustrate one way these coefficients may be used, a diatom-based transfer function was developed for paleolimnological applications. Transfer function evaluation and testing typically involve the comparison of algal-inferred water quality to measured water quality to evaluate function robustness, which is usually characterized by a coefficient of determination (r^2^) and a prediction error. It is well-known that adjacent phytoplankton samples (e.g. 5–100 km apart) from the Great Lakes tend to have similar species assemblages and environmental conditions, and this lack of independence among sites can violate statistical assumptions. Such autocorrelation may result in misleading estimates of transfer function performance [21], and with new analyses revealing previously undocumented weaknesses with diatom-based nutrient models [22], it is imperative that transfer functions undergo thorough testing. So, we evaluated how (1) autocorrelation, (2) relationships between taxon abundance and phosphorus and (3) redundancies among environmental variables affected our training set and its predictive power. We further tested the model by applying it to fossil diatom assemblages from Lake Superior to determine whether pelagic diatom-inferred phosphorus serves as an effective paleoecological tool in the Great Lakes.

## Materials and Methods

We evaluated phytoplankton data collected as part of the USEPA's biological monitoring program for the Great Lakes. The standard operating procedure for phytoplankton collection and analysis is described in detail in the published procedures [11], but abbreviated details were as follows. The EPA data were based on twice-annual synoptic sampling (“spring” = typically the month of April, “summer” = typically the month of August) from standard stations throughout the Great Lakes basin. Our analyses focused on phytoplankton and water quality samples collected from 2007 through 2011, a total of 717 unique sampling events. No specific permissions were required for these locations or activities; namely, sample locations in the pelagic Great Lakes. Field studies did not involve endangered or protected species.

Whole water samples were collected from the rosette sampler on-board the Research Vessel Lake Guardian. Phytoplankton samples were composites of water sampled at discrete depths from the euphotic zone of the water column. For isothermal spring samples, the sample integrated equal volumes of water from 1, 5, 10 and 20 m. In shallower locations in Lake Erie the 20-m sample was replaced by an above-bottom collection. If the total depth was less than 15 m, equal volumes were integrated from surface, mid-depth and above-bottom samples. For the stratified (summer) water column, equal volumes were taken from 1 m, 5 m, 10 m and the lower epilimnion and integrated. If the epilimnion was very shallow, equal volumes were integrated from a maximum of four and a minimum of two sampling depths. Samples were split and analyzed separately for the whole phytoplankton assemblage (i.e., “soft” algae) and diatoms. Analysis of soft algae used the quantitative Utermöhl method of counting preserved specimens in a settling chamber on an inverted microscope [23]). During soft algal analyses, diatoms containing cytoplasm were identified as centric or pennate forms. The second split sample was digested in nitric acid and subsequently in peroxide to isolate the diatom valves which were then plated on slides and counted using oil immersion (1000× or higher) to identify taxa. All counting included measurements of cell dimensions so that algal biovolumes could be calculated. Ultimately, analyses afforded detailed taxonomic resolution, and data were available in quantitative data formats (cell density [cells/ml] and biovolume [µm^3^/ml]).

A 30-cm sediment core from eastern Lake Superior was used as a test subject for a diatom-based phosphorus transfer function. Details of sediment coring and sample preparation are provided by Shaw Chraïbi et al. [24]. The stratigraphic record of diatom assemblages dating back to the early 1700s was the subject of testing in the present assessment.

**Figure 1:**
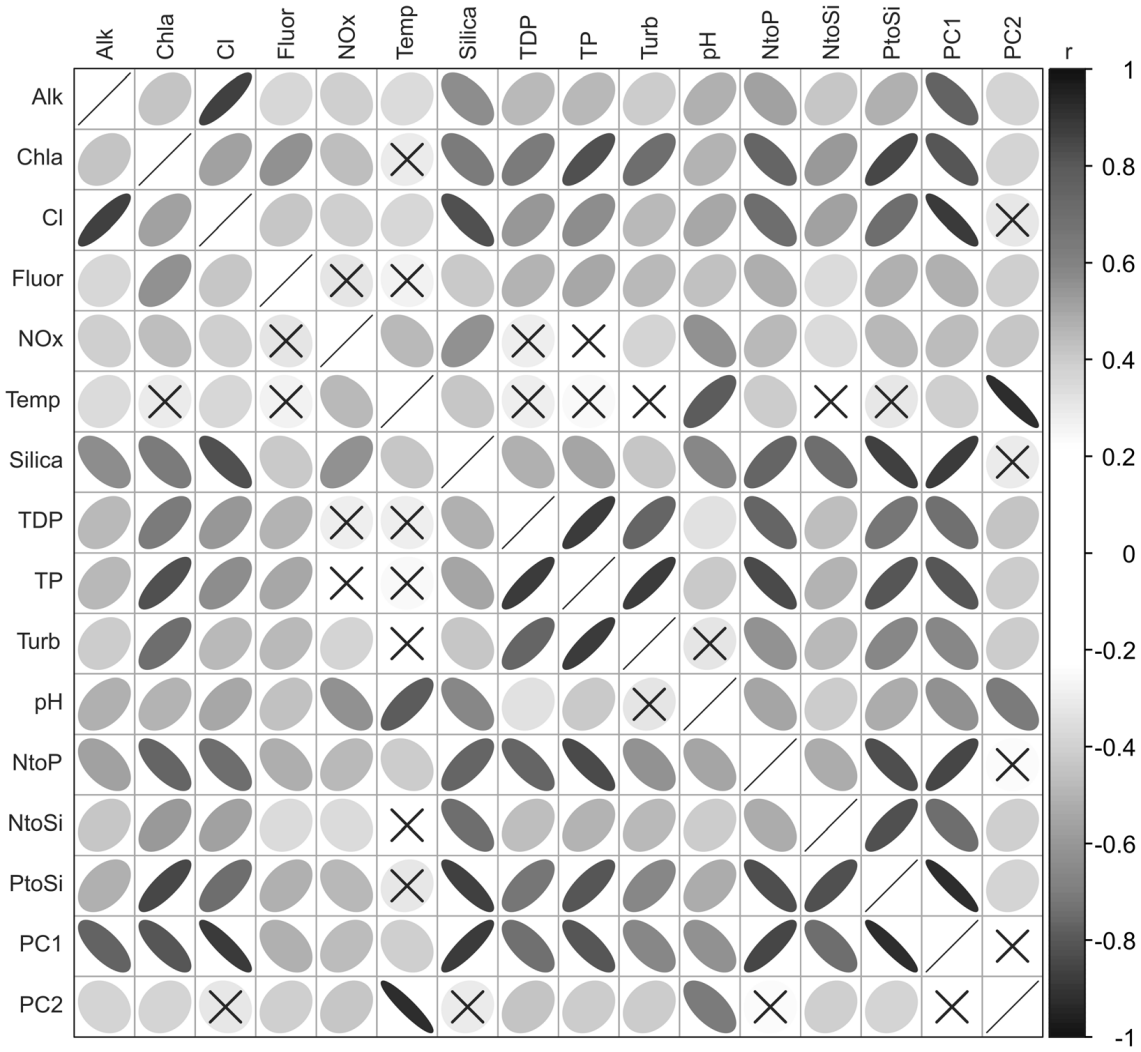
Correlation matrix of Great Lakes water quality variables. Correlation matrix of Great Lakes water quality data, using Pearson product-moment correlation coefficients. Ellipses summarize positive or negative correlations, with narrower, darker ellipses indicating stronger correlations. Variable pairs containing an × were not significant (P = 0.05 with Bonferroni correction for multiple comparisons). All variables were transformed as necessary to minimize skew and approximate or achieve normality (Table 1). Scores from the first two axes of a principal components analysis are included to indicate important variables for these gradients.

Algal associations with 11 environmental variables (Table 1), collected simultaneously with phytoplankton, were explored. Additional variables were considered but were redundant (specific conductivity with chloride, beam attenuation with turbidity) or poorly represented in the dataset (dissolved oxygen, irradiance, total nitrogen). Geophysical variables (e.g., depth, latitude, longitude) were not used in multivariate analyses in order to better describe relationships with water quality, although the importance of “lake” as a nominal variable was explored in terms of species specificity. Collection and analysis of environmental data is described in detail in the USEPA (2010) standard operating procedures. Three additional molar ratio variables (N∶P, N∶Si, P∶Si) were calculated from other variables in the list so that they may be used in the evaluation of algal responses across nutrient ratio gradients. Because of substantial correlation among these variables (Figure 1), multivariate approaches were deemed necessary to determine species-environmental relationships.

**Table 1:** The environmental variables used in this study.

Variable	Abbreviation	Transformation	Minimum	5th percentile	Median	Mean	95th percentile	Maximum
Alkalinity (mg/L)	Alk	sqrt	41.90	43.00	86.00	78.40	109.00	124.00
Chlorophyll a (µg/L)	Chla	log	0.00	0.44	1.03	2.54	10.12	35.39
Chloride (mg/L)	Cl	sqrt	1.20	1.29	11.57	10.39	22.69	29.27
Fluorescence (µg/L)	Fluor	log	0.00	0.11	1.39	2.41	7.53	50.96
Nitrates + Nitrites (mg/L)	NOx	sqrt	0.00	0.08	0.32	0.30	0.43	1.63
Temperature (°C)	Temp	sqrt	0.11	1.02	7.19	11.23	24.99	26.81
Silica (mg/L)	Silica	-	0.00	0.02	0.82	0.69	1.11	1.63
Dissolved Phosphorus (µg/L)	TDP	log	0.45	0.85	1.90	2.75	6.40	69.70
Total Phosphorus (µg/L)	TP	log	0.72	1.54	2.96	6.26	18.08	226.78
Turbidity (NTU)	Turb	log	0.11	0.16	0.30	1.64	5.23	178.00
pH	pH	-	7.58	7.89	8.31	8.33	8.80	9.20
N∶P (molar ratio)	NtoP	sqrt	0.00	0.01	0.10	0.10	0.22	0.37
N∶Si (molar ratio)	NtoSi	log	0.00	0.29	0.37	1.49	5.74	120.95
P∶Si (molar ratio)	PtoSi	log	0.98	1.58	3.37	84.48	414.08	3702.76
Principal Component 1	PC1	-	−0.91	−0.68	0.12	0.00	0.46	0.58
Principal Component 2	PC2	-	−0.63	−0.52	0.04	0.00	0.50	2.29

Provided are untransformed ranges of the variables and the transformations applied to remove skew. A principal components analysis was performed to derive the summary axes (PCs).

All analyses were performed using the R statistical software [25] and associated packages. All scripts and datasets are available from the authors on request. Phytoplankton and water quality data are archived in the Great Lakes Environmental Database (http://www.epa.gov/greatlakes/monitoring/data_proj/glenda), and the paleoecological data are provided electronically in Shaw Chraïbi [26].

### Ordination

Multivariate ordinations were performed using the R package “vegan” [27]. An initial principal components analysis (PCA) was used to characterize the major environmental gradients in the water quality data. All environmental variables were scaled to zero mean and unit variance. The first two principal components (axes), which are a linear combination of the greatest variation in environmental condition across sites, were used as new environmental variables (Figure 1) in the exploration of algal species autecologies.

For diatom-environmental analyses, redundancy analysis (RDA), the constrained form of PCA, was performed to condense the complex dataset into summary variables (axes) that capture the majority of environmental variation (Juggins and Birks [28]). An RDA with a Monte Carlo permutation test was used to identify the environmental variables with significant (P<0.05) relationships with the diatom data. The subset of significant variables was then selected to generate a new RDA to identify species-environmental relationships and orthogonal gradients that capture variation in the diatom data. Explained variation from this RDA was compared to a partial RDA that partitioned variation in the diatom data by characterizing the total and unique contributions that can be attributed to TP. This was further applied to the variables alkalinity (Alk), chloride (Cl) and nitrates + nitrites (NOx) to examine similar characteristics for variables that may be confounded with TP (e.g., Alk, NOx) or have strong independent controls on diatom autecology (e.g, Cl).

### Species-environmental relationships

Algae-based inference models typically assume the taxa respond unimodally across the environmental gradient of interest [29]; however, based on many possible factors (e.g., narrow gradients, multivariate controls, inadequate sampling or unique ecological distributions) taxa may vary in their responses, or have no apparent response. It is important to characterize the goodness of fit of species to the environmental variable, as well as to consider linear, Gaussian or skewed responses along gradients in order to validate the assumption that diatom species responses actually reflect the environmental variable of interest.

Responses of algae taxa along environmental gradients were evaluated using linear, Gaussian and weighted-average (WA) assumptions. First, environmental variables were transformed as necessary to minimize skew and achieve normality in their frequency distribution, if possible. Taxa that were observed in more than five samples were evaluated. Responses were evaluated using four measures of algal abundance: cell densities (cells/ml), biovolume (µm^3^/ml), relative cell density and relative biovolume.

Using the “stats” (version 3.0.0) package [25] the function “lm” was used to generate a linear model, then the function “cor.test” was used to determine linear model relationships between taxon abundance and each environmental variable. Significance (P = 0.05) was tested based on Pearson's product moment correlation coefficient.

Using the “gam” (version 1.08) package [30], the “gam” function was used to fit Gaussian model relationships between taxon abundance and each environmental variable. Based on a comparison of residuals, a paired *t*-test (P = 0.05) was applied to determine whether a Gaussian fit was better than a linear fit. The point on the environmental gradient where a Gaussian fit was maximized was considered a taxon's optimum. The environmental weighted-average optimum for a taxon was calculated based on the average of environmental values where the taxon occurred, weighted by taxon abundance. Taxon optima based on Gaussian and WA calculations were rescaled to a range of 0 to 10, which was based on the range of the transformed (if applied) environmental variable. This rescaling resulted in more user-friendly coefficients (i.e. “environmental indicator values”), but coefficients based on actual variable units are available from the authors.

To support the utility of taxa included in the diatom-based transfer function, generalized additive models (GAMs [31]) were used to test the significance of the response of each diatom taxon to the measured TP gradient using the R package “mgcv” [32]. The additive models test several response forms (linear, unimodal, skewed) across the selected gradient. We further tested the significance of the unique explanatory power of TP once the effect of alkalinity (another important water quality variable in the Great lakes) was removed. GAMs were tested on the relative density data for taxa with 10 or more occurrences in samples and significance of fitted models was assessed using 199 Monte Carlo permutations of the deviance explained by TP for each species response GAM. (Although a less stringent criterion for occurrence [five samples] was used in the development of indicator values, here we use 10 samples to ensure sufficient abundance to identify responses along the TP gradient.) Data were evaluated based on relative density and relative biovolume as these are the data types that may be used in diatom-based paleoecological studies.

### Diatom-based transfer functions

Transfer functions for Great Lakes total phosphorus (TP) were derived using the diatom components of the algal assemblages. As such a function is most likely to be used for down-core analyses in paleolimnological assessments [29], the soft algae that are less likely to preserve in the sedimentary record were not used. Transfer functions were developed using weighted averaging (WA) regression with inverse de-shrinking with log-normal taxa transformation (“rioja” package [33]). Diatom-inferred estimates of TP (DI-TP) for each sample were calculated by taking the optimum of each taxon to that variable, weighting it by its abundance in that sample, and calculating the average of the combined weighted taxa optima ([34], [35]). The apparent strengths of the transfer functions were evaluated by calculating the squared correlation coefficient (r^2^) and the root mean square error of prediction (RMSEP) between measured TP and transfer function estimates of those values for all samples. The predictive error was estimated by jackknifing [36] which corrects for under-estimation of root-mean squared error due to predicting values only from samples that were included in the model. As above, variations of these functions were tested using two possible representations of the algal data, relative cell density and relative biovolume. Developing such functions based on absolute abundances was not deemed useful for future applications because species abundances in sedimentary assemblages (e.g., cells per g dry sediment) cannot be assumed to be comparable to prevailing phytoplankton concentrations (e.g., cells per mL in the water column).

### Transfer function testing

We evaluated redundancy in the training set using the modern analog technique (MAT; [37]) with five analogs. Instead of using the weighted abundance of taxa, a reconstructed value was based on the five most taxonomically similar samples in the training set. The effect of autocorrelation on transfer function performance was evaluated using the “rne” function in the R package “palaeoSig” [38]. The rne function compares the performance (r^2^) of the transfer function during cross-validation as sites are deleted (1) randomly, (2) that are geographically close to the test site, and (3) that are environmentally similar to the test site [21]. Reducing the number of sites will worsen the performance as the number of potential analogues decreases [39]. In the case of autocorrelation, deleting the nearest sites would remove the best analogues, and should be more detrimental to performance than random deletion. Such degradation would also be expected when sites in close environmental proximity (e.g., with similar TP) are selectively deleted. If performance deteriorates more by deletion of close sites than by deletion of environmental neighbors, the performance loss relative to the random deletion must in part be due to spatial autocorrelation between diatom assemblages.

We further checked the diatom-TP transfer function performance against several randomized simulations using multiple iterations of the “multi.mat” function in palaeoSig [21]. This method simulates the environmental variable across sites using the same autocorrelation structure as the measured data and recalculates performance (r^2^). Creating simulated environmental data requires many geographical considerations, including development of an empirical variogram to determine the spatial structure of TP in the Great Lakes, fitting a theoretical variogram model, and a simulation to generate a spatially structured random variable with the same spatial structure as the original data. We employed the gstat package [40] in R to support these calculations, and the method is detailed by Telford and Birks [21]. A transfer function is considered to have statistically significant predictive power (p<0.05) if the real value of r^2^ for the transfer function exceeds 95% of the simulations.

### Application to paleolimnology

Reconstructed estimates of past TP concentrations were obtained from sedimentary samples collected as part of a recent paleolimnological analysis of eastern Lake Superior (Shaw Chraïbi et al. [24]). Despite the training set samples representing seasonal snapshots of modern diatom assemblages as opposed to sedimentary assemblages containing year-round integrations of settling diatoms, paleo-assemblages had several good modern analogues in the training set, thus providing preliminary evidence that the training set may be applicable (Shaw Chraïbi [26]). Diatom-inferred TP from the uppermost sediment samples matched well with contemporary TP monitoring data (Shaw Chraïbi et al. [24]). Downcore TP estimates were calculated using WA with inverse de-shrinking (Juggins and Birks [28]). To track the historical trajectory in relation to historical diatom conditions, sedimentary assemblages were projected on the RDA containing the suite of training set diatom data and significant environmental variables.

We then calculated the ratio of two values: (1) λ_R_, the variance in sedimentary diatom assemblages captured by the first axis of a RDA constrained to DI-TP; (2) λ_P_, the variance explained by the first axis of an unconstrained PCA of the diatom data. The λ_R_/λ_P_ ratio expresses the proportion of variation explained by DI-TP as a fraction of the maximum explainable variance in the sedimentary samples. We also calculated the correlation coefficient for past DI-TP versus corresponding axis 1 sample scores from the unconstrained PCA of the sedimentary diatom assemblages. We would expect both of these values to be high (i.e. close to a proportion of 1.0) if downcore assemblages were strongly related to changes in TP [22].

## Results and Discussion

### Taxon-specific results

263 taxa were sufficiently abundant (i.e. in more than five samples) to be considered for evaluation along environmental gradients. The common taxa comprised centric diatoms (42 taxa), pennate diatoms (103 taxa), chrysophytes (37 taxa), green algae (45 taxa), cryptomonads (8 taxa), blue-green algae (17 taxa), euglenoids (2 taxa), dinoflagellates (6 taxa) and some unknown entities (3 taxonomic categories). Some were unknown species or genera not identifiable to the species level (a genus followed by “spp.”) and others were members of known divisions but with few diagnostic characteristics (e.g., “unidentifiable chrysophyte ovoid”). Although these less specific taxonomic categories are difficult to understand ecologically due to the likelihood of multiple species comprising a category, we present them as possible inference tools because several of them had significant relationships with environmental variables.

Table 2 presents environmental characteristics for select taxa relative to nine variables, as well as their lake and seasonal specificity, based on observed algal biovolumes and relative cell densities in phytoplankton samples. It is beyond the scope of this article to characterize the numerous species-specific mechanisms for these environmental relationships, but some of these taxa are recognized as important components of the Great Lakes phytoplankton community. The full suite of species coefficients are provided in supplementary appendices that detail species coefficients based on cell densities (Table S1), relative cell density (Table S2) biovolume (Table S3) and relative biovolume (Table S4). These tables further characterize within-lake conditions and may serve as a set of coefficients suited to further indicator development and monitoring assessments.

**Table 2:** Environmental indicator values for select algae taxa in the Great Lakes.

	Season		Lake					Chlorophyll a			Chloride			Fluorescence			Nitrates + Nitrites			Temperature			Silica			Total Phosphorus			pH			N∶P		
Taxon Full Name	Spring	Summer	Superior	Michigan	Huron	Erie	Ontario	Lin	Gau	WA	Lin	Gau	WA	Lin	Gau	WA	Lin	Gau	WA	Lin	Gau	WA	Lin	Gau	WA	Lin	Gau	WA	Lin	Gau	WA	Lin	Gau	WA
*Aulacoseira distans* (Ehr.) Simonsen	9	1	1	7	1	1	0	+	3*	2	+	5*	5		7	2		10	3	+	3*	3	−	10*	6	+	10*	2		3	3	−	3	3
*Aulacoseira granulata* (Ehr.) Simonsen	0	10	0	0	0	10	0	+	10*	8		6	6	+	8	6		0*	1	+	10	10		3	4		7	5	+	10	8		0	0
*Aulacoseira islandica* (O. Mull.) Simonsen	10	0	0	0	0	10	0	+	10*	7	+	7*	7	+	10*	5	−	0*	1		3	2	−	0*	0	+	5*	5	+	6*	5	−	0*	0
*Cyclotella michiganiana* Skv.	0	10	0	7	2	0	0		0	2	+	5	5		0	1	−	2*	2	+	9*	8	−	4*	4		0	1	+	6*	6	−	3	3
*Fragilaria crotonensis* Kitton	0	10	0	1	0	8	2	+	6	5	+	7*	7	+	6	4	−	0*	2	+	10*	9	−	1*	2	+	4	3	+	10*	7	−	1*	1
*Cryptomonas erosa* Ehr.	3	7	1	1	0	6	2	+	6	4	+	7*	6	+	4	3	−	0*	2	+	10*	7	−	0	3	+	5	3	+	10*	6	−	0*	1
*Cryptomonas reflexa* Skuja	2	8	1	1	0	6	2	+	6	4	+	6*	6	+	4*	3	−	0*	2	+	10*	8	−	4	3	+	5	3	+	10*	6	−	0*	1
*Cryptomonas rostratiformis* Skuja	3	7	4	0	0	2	4		6	3	+	10*	4		1*	2	−	1	2	+	8*	6	−	0*	4		3	2	+	0*	5		10	3
*Microcystis aeruginosa* (Kütz.) emend. Elenkin	0	10	0	0	0	10	0		10	9		6*	6	+	8	6		0*	1	+	10*	10		3*	4	+	7*	6		10	9		0*	0

Select algae taxa in the Great Lakes and their environmental indicator values based on biovolume distributions across the environmental gradients. Negative (−) and positive (+) signs indicate significant linear relationships (P<0.05) based on the Pearson product moment correlation. Gaussian optima (Gau) display an asterisk (*) if fitted residuals were significantly smaller (t-test; P<0.05) than residuals from the linear fit. WA indicates optima based on weighted-averaging. “Season” and “Lake” coefficients reflect seasonal and spatial occurrence of the taxa. Indicator values from 0 through 10 respectively represent whether a given taxon had its greatest modeled abundance at a low or high region of a variable's gradient. Indicator numbers for seasons or lakes represent the relative proportion that each taxon achieved in 2007–2011, again scaled from 0 to 10.

Several taxa have high (e.g., *Aulacoseira distans*) or low (e.g., *Cyclotella michiganiana*) optima for TP. However, it is interesting that all of the significant linear species relationships with TP are positive (Table 2, Table S3). A similar negative effect occurred for silica. This appears to be due to the dominating effect of summer in Lake Erie, a time when TP is high, silica is low, and several algal taxa are highly abundant, thus driving positive slopes in the biovolume-TP relationship. A comparative examination of relative densities (Table S2) indicates a greater diversity in species responses to TP because relative abundances were more evenly distributed across lakes and environmental gradients.

Based on weighted abundance across all samples, the centric diatom *Aulacoseira islandica* is the most abundant alga in the Great Lakes phytoplankton ([41]). However, it has a very specific spring bloom period in Lake Erie, with very low occurrence across the other lakes. Its distribution reflects high chlorophyll *a* and fluorescence, which is not surprising as it comprises most of Lake Erie's spring algal bloom. *A. islandica* has a significantly negative relationship with silica, which seemingly counters its very high silica requirement in the formation of its heavily silicified cell walls. This may imply that by the time spring sampling occurs in April of each year diatoms have exhausted the dissolved silica in the water that is available to incorporate into their cell walls.

While structurally similar to *A. islandica*, *Aulacoseira granulata* is a distinctly summer diatom in Lake Erie. *A. granulata* has an apparent tolerance for warmer, late-summer conditions when it coexists with green and blue-green algae [42] that account for high chlorophyll concentrations. *Fragilaria crotonensis* is a pennate diatom that also occurs in the summer in Lake Erie, with lower abundances in Ontario and Michigan. It has high optima for pH and temperature and appears to be associated with a low N∶P ratio.

The cyanophyte *Microcystis aeruginosa* is a well-known coastal bloom species in Lake Erie [43], and it has been observed abundantly in recent offshore phytoplankton samples [42]. Our assessment confirms its fairly strict occurrence in the summer in Lake Erie, as well as its occurrence at times of high chlorophyll *a*, temperature and pH, as well as low nitrates and N∶P.

Three cryptomonad algae were abundant: *Cryptomonas rostratiformis*, *C. reflexa* and *C. erosa*. These species occurred year-round in all of the lakes. While seemingly cosmopolitan, there were clear distinctions within this genus, such as *C. reflexa* being tolerant of high chloride and temperature while *C. rostratiformis* occurred under conditions of low chloride and temperature.

### The diatom-based model

Under the full diatom-inferred TP model, optimal model parameters included use of WA with tolerance down-weighting, which offered minimal improvement. The model was developed using log-transformation of the TP data and it was cross-validated using the jackknifing procedure [35]. There were no transformations of the taxonomic data or removal of taxa or samples as these procedures did not improve apparent model performance. We tested removal of benthic and rare taxa (i.e., occurring in fewer than 5 samples) but such considerations also degraded model performance. Comparisons between observed TP and DI-TP resulted in a jackknifed r^2^ of 0.79 and RMSEP of 0.38 (log-TP units) using relative density data and an r^2^ of 0.77 and RMSEP of 0.36 using relative biovolumes (Table 3).

**Table 3:** Results of DI-TP model testing using diatom data based on relative cell densities and biovolumes.

	Density	Biovolume
Model r^2^_jackknife_	0.79	0.77
Model RMSEP (log-TP units)	0.38	0.36
Number of taxa with significant GAM relationship to TP (out of 118)	90	89
Number of taxa with significant relationship to TP after factoring out Alk (out of 118)	65	67
Percentile of actual model in simulated remapping of training set	99.8%	99.8%
RDA 1 eigenvalue	16.7%	20.0%
RDA 2 eigenvalue	6.4%	7.4%
Total variance explained by TP	6.8%	9.1%
Unique variance explained by TP	4.8%	6.0%
Downcore DI-TP versus PCA 1 r	0.98	0.68

Based on GAM analyses of relative density data for each common diatom taxon, 90 out of 118 taxa (76%) taxa had a significant response across the TP gradient. After removing the effects of alkalinity, 65 out of 118 (55%) had a significant response to TP, indicating that 25 of the taxa with significant relationships with TP had a signal that was confounded with alkalinity. Comparable results using relative biovolumes revealed 89 taxa with significant responses to phosphorus, 67 after accounting for alkalinity. Basing the model on diatom densities or biovolumes appeared to have little effect on model performance. It is more typical in paleolimnological studies to use count data without the additional benefit of cell measurements to calculate biovolumes, so subsequent figures focus on the use of relative diatom valve densities.

**Figure 2:**
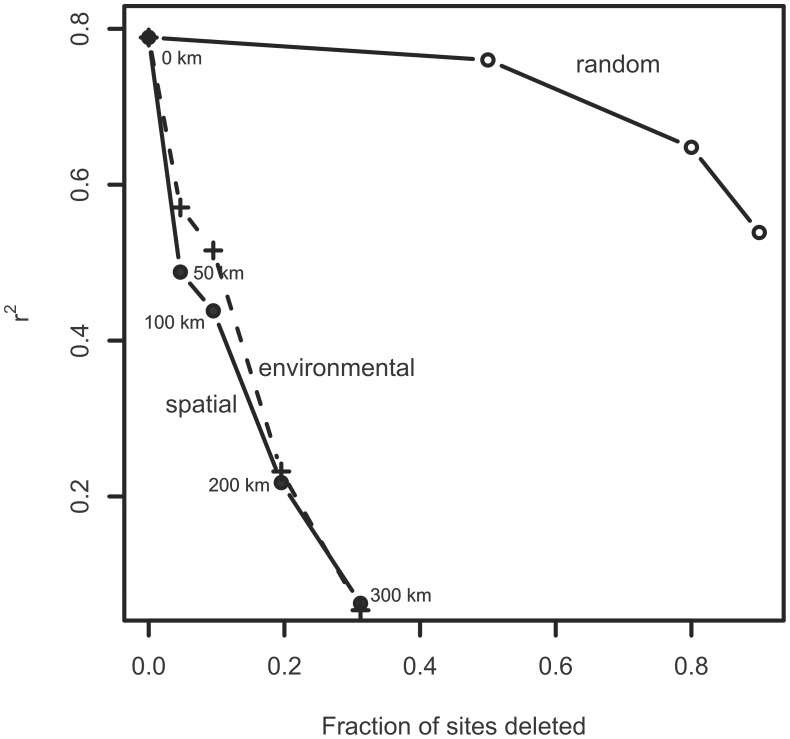
Effect of deleting sites on diatom-TP model performance. The effect on transfer function r^2^ of deleting sites at random (open circles), from the geographical neighborhood of the test site (filled circles), and those that are environmentally similar (crosses) during cross-validation of the Great Lakes diatom training set. The radius of each distance is labelled.

In a check of the effects of sample removal in the training set, random removal of samples had a small effect on apparent model performance (Figure 2). For instance, the observed-inferred r^2^ decreased from 0.77 for the full model to 0.66 after removal of 80% of samples. However, removal of spatially and environmentally similar sites had a more substantial impact on performance, indicating autocorrelation among sites. Removal of sites that were geographically close to the test site in cross-validation resulted in a sudden drop in the r^2^, as low as 0.13 after sites within 300 km were removed. This result is not surprising given the known uniqueness of the phytoplankton assemblages in each lake [44], so removal of training set samples in the same lake as the test site undoubtedly had a substantial effect on assemblage analogs. A similarly precipitous drop in performance occurred due to removal of sites that were environmentally similar to the test site, but model degradation was not noticeably different from that due to spatial removal. The relative importance of geographical and environmental neighbors shows how important adjacent sites are for the performance of the transfer function. As explained by Telford and Birks [21], the occurrence of autocorrelation indicates potential problems in a transfer function, and so the modern analog technique (MAT [37]) of inference is not recommended for this model. However, the model may still have predictive power under weighted-averaging scenarios. Based on models derived by random re-mapping of the environmental data over the species map, the actual model performed better 99.8% of the time (Table 3; Figure S1), indicating that the diatom-TP WA transfer function is statistically significant, suggesting that TP can be reconstructed from this training set.

**Figure 3:**
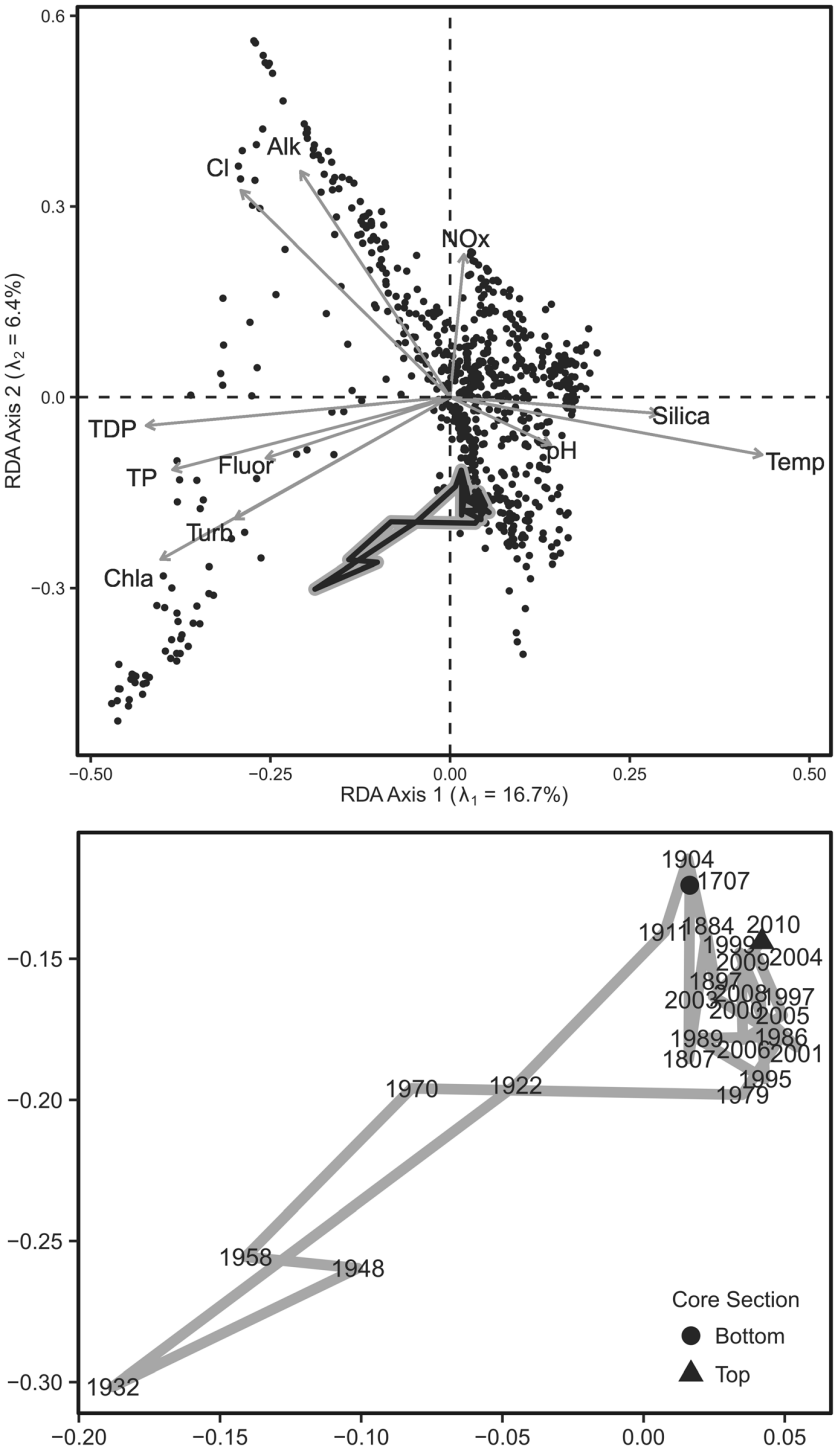
Redundancy analysis of the diatom training set constrained to significant water quality variables. Eigenvalues indicate variance explained by each axis. Passive plotting of the Lake Superior sedimentary assemblages is indicated by the black line set in a thicker gray line. The lower panel is a zoomed-in plot of the passive ordination of Lake Superior sedimentary diatom assemblages. The core top (2010) and bottom (∼1707) are indicated and the date of each interval (based on ^210^Pb dating) is provided for each interval.

An RDA of the diatom assemblages constrained by the 11 significant environmental variables indicated that these variables account for a major part (16.7% on the first axis; Table 3, Figure 3) of the underlying gradients in the diatom relative density data. Axis 1 of an unconstrained PCA captured 20.6% of the variation in the diatom data, indicating that the significant environmental variables accounted for a large portion of the underlying species gradients. Environmental data accounted for a larger portion of the variance in diatom relative biovolume data (20.0%; Table 3). TP (and the related TDP) were strongly correlated to axis 1, the main gradient of species turnover. There is also a clear alkalinity/chloride gradient that diagonally represents axes 1 and 2. Axis 2 captures a smaller proportion of variance (6.4%), although it is closely correlated to the NOx gradient. These proportions of explained variance are typical and informative in multivariate analyses of biological abundance data that tend to be noisy (Gauch [45]).

**Figure 4:**
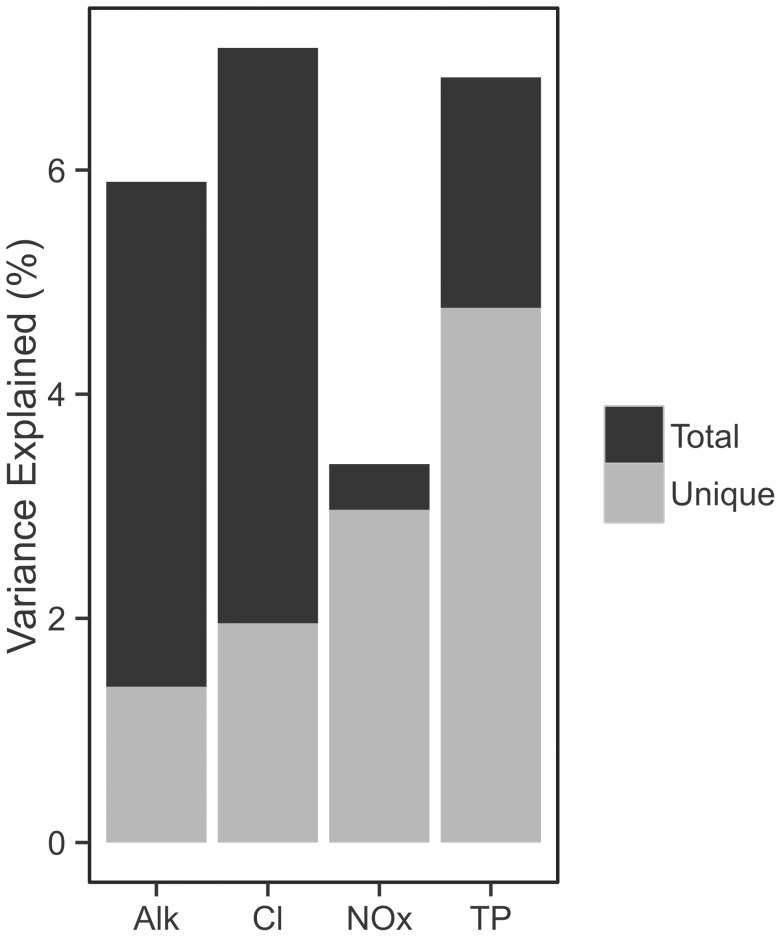
Histograms show percentage of variation in data explained by four water quality gradients. Total height indicates the total variation explained while gray height indicates the unique fraction of variance captured by that variable.

Figure 4 illustrates variance partitioning of the diatom relative density data by selected environmental variables. The total and unique effects of TP were 6.8% and 4.8% respectively (Table 3), a 29% reduction in explained variance. Based on relative biovolumes the total and unique effects were 9.1% and 6.0%, indicating a closer linkage between TP and diatom biovolumes than densities. That the unique TP effect is lower than total is due to redundancies, such as correlation to variables like chlorophyll *a* and turbidity (Figure 1). While it is difficult to confirm the relative importance of these variance partitioning values, the unique variance explained by TP surpasses that observed for TP training sets developed in Europe [46] and Minnesota [47], [48]; 3.9% and 2.5% respectively, as presented by Juggins et al. [22]. Of the selected variables, TP independently explained the most variation in the assemblage data, in contrast to alkalinity and chloride which had more than 70% reduction from total to uniquely explained variance. NOx explained the least variation but was minimally confounded by other variables. For TP to be confounded with other correlated variables is not surprising, but these results suggest that changes in the diatom assemblages across the Great Lakes are meaningfully related to TP.

### Downcore testing of the diatom-based model

**Figure 5:**
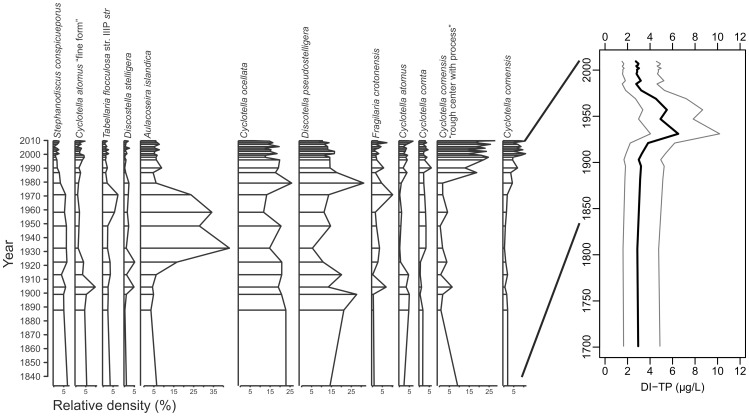
Dominant diatom species (>5% relative abundance) for the eastern core of Lake Superior. A plot of corresponding DI-TP for the fossil assemblages is shown on the right. Black line indicates inferred TP and grey lines indicate the range of model error (RMSEP).

Figure 5 presents the common diatom taxa (>5% in any sample) in a sediment core from eastern Lake Superior (unpublished data). The stratigraphy is based on relative diatom density, including the DI-TP results which indicate a temporary enrichment event. This inference was largely driven by a short-term abundance of *A. islandica* which temporarily replaces lower-nutrient *Cyclotella* and *Discostella* species through much of the 20th century. Passively plotting the Lake Superior fossil samples on the RDA traces the path of historical changes in the diatom assemblages (Figure 3). The long-term trend indicates a temporary movement of the scores in the direction of the TP vector from 1922 through 1970, a period that reflects nutrient enrichment in Lake Superior in association with the increased abundance of *Aulacoseira islandica* (unpublished data; [49]). Subsequent to 1970 the sample scores revert to locations close to pre-Euro-American samples, indicating TP reductions as the diatom assemblage shifted to a greater dominance by *Cyclotella* (unpublished data).

There was a high correlation (r = 0.98; N = 26) between DI-TP and the main gradient in the Lake Superior sedimentary diatom data (Figure S2), indicating TP was a strong driver of Superior's assemblages. The fraction of variance explained by DI-TP, represented as a fraction of the maximum explainable variance, was also high (λ_R_/λ_P_ = 0.97). These results suggest that phosphorus has been an important determinant of diatom assemblages in Lake Superior, and that the TP transfer function was well-suited to reconstructing the nutrient history of the lake.

## General Discussion

We provide a suite of environmental coefficients for the dominant phytoplankton taxa in the Laurentian Great Lakes. We hope that these data increase understanding of taxon-specific ecology, and expect these data will be used to develop inference models such as the diatom-based model tested herein. We endorse using these species coefficient tables (Table 2, Tables S1, S2, S3, S4) as checklists for future indicator studies. For instance, Kelly and Whitton [50] provided a checklist of trophic coefficients for diatom specimens, calibrated to nutrient and organic pollution variables, from rivers in the United Kingdom. Their coefficients have been used repeatedly in ecological assessments intended to manage river ecosystems [51]. Model-specific coefficients (linear, Gaussian, WA) and their statistical significance may also be used selectively in development of an indicator.

We recommend some cautions in using these data to infer environmental conditions. The coefficients are taxon-environmental relationships based on simultaneous sampling of algae and water quality. A phytoplankton assemblage may be a product of water quality conditions prior to sampling. For instance, despite the low silica optimum for *Aulacoseira islandica*, we know this taxon requires high silica concentrations for cell wall development [52], and so the Lake Erie spring diatom assemblages are undoubtedly a product of high silica concentrations prior to spring sampling. So, while high densities of *A. islandica* may infer low dissolved silica, the taxon may not represent a prevailing condition of low silica. We expect coefficients based on water quality variables that were not limiting at the time of sampling (e.g., spring nutrients [53]) are more reliable.

The provided transfer function, a diatom-based total phosphorus model, shows promise for future applications in the Great Lakes. Although internal testing of the model showed substantial spatial and environmental autocorrelation among sample locations, which is to be expected in large lakes that are likely homogeneous across vast areas, further testing indicates that phosphorus is an important driver of diatom assemblages in the Great Lakes, and that paleolimnological studies may benefit from this indicator model.

Unlike the snapshot approach of phytoplankton sampling, diatom assemblages retrieved from sediment cores reflect year-round integration of valves. For our Lake Superior example we confirm good analogues between fossil and training set assemblages, as well as a credible long-term TP reconstruction. Past assemblages, which included relatively high abundances of *A. islandica* during the mid-20th century, differ greatly from the phytoplankton in Lake Superior today, so the TP reconstruction relied on taxon coefficients that were largely derived from other lakes that presently contain abundant *A. islandica* (e.g., Lake Erie). It is unknown whether we may assume similar performance from cores collected elsewhere in the Great Lakes system, so we recommend thorough downcore validation of the model as we have done here, and as others recommend [22], before making conclusions about paleoecological nutrient trends. Also, the reconstructed range of TP for Lake Superior was on the low end of the range for the Great Lakes as a whole, so future testing should consider whether reconstructions in more nutrient-rich lakes are possible.

Lake Superior has encountered substantial diatom assemblage reorganization in the last 200 years, and as speculated by others [49] these changes were largely driven by anthropogenic nutrients (i.e. the rise and fall of pollutant flux to the lake). A sedimentary record containing similarly strong historical revisions in assemblages should have a strong assemblage-DI relationship as we observed for TP in Lake Superior, otherwise an investigator must explore other variables as the cause of historical shifts. However, monotonous stratigraphic records of diatoms will have narrow ecological breadth, and a probable narrow range of DI-TP. Such cases may result in a weak correlation between assemblage patterns and DI data, so other means would be necessary to confirm the importance of TP to the sedimentary record.

In conclusion, the taxon-specific autecological information provided herein may be used to inform future observations of these species within the Great Lakes and elsewhere. More importantly, Great Lakes phytoplankton assemblages can make a contribution to indicator studies as suggested by Stoermer [10]. We present one of many indicator possibilities; the transfer function presented here appears to be suitable for downcore reconstructions. Using similar rule-based models with clear validation protocols should lead to refined, defensible environmental predictions and reconstructions.

## Supporting Information

Figure S1 **Performance of 1000 transfer functions with simulated environmental variables based on re-mapping of the TP dataset.** 998 simulated transfer functions had poorer performance than the actual model r^2^ based on the observed-inferred TP relationship, as indicated by the dotted line.
(TIF)

Figure S2 **Relationship between Lake Superior downcore DI-TP and PCA axis 1 for diatom data.**
(TIF)

Table S1 **Environmental indicator values for the common algae taxa (>5%) in the Great Lakes based on distribution of cell densities across the environmental gradients.**
(XLSM)

Table S2 **Environmental indicator values for the common algae taxa (>5%) in the Great Lakes based on distribution of relative cell densities across the environmental gradients.**
(XLSM)

Table S3 **Environmental indicator values for the common algae taxa (>5%) in the Great Lakes based on biovolume distribution across the environmental gradients.**
(XLSM)

Table S4 **Environmental indicator values for the common algae taxa (>5%) in the Great Lakes based on distribution of relative biovolume across the environmental gradients.**
(XLSM)
