# Supplementary figures and images for: Laurentian Great Lakes Phytoplankton and Their Water Quality Characteristics, Including a Diatom-Based Model for Paleoreconstruction of Phosphorus
Source: PLoS One. 2014 Aug 8;9(8):e104705. doi: 10.1371/journal.pone.0104705 (PMC4126776; doi:10.1371/journal.pone.0104705)

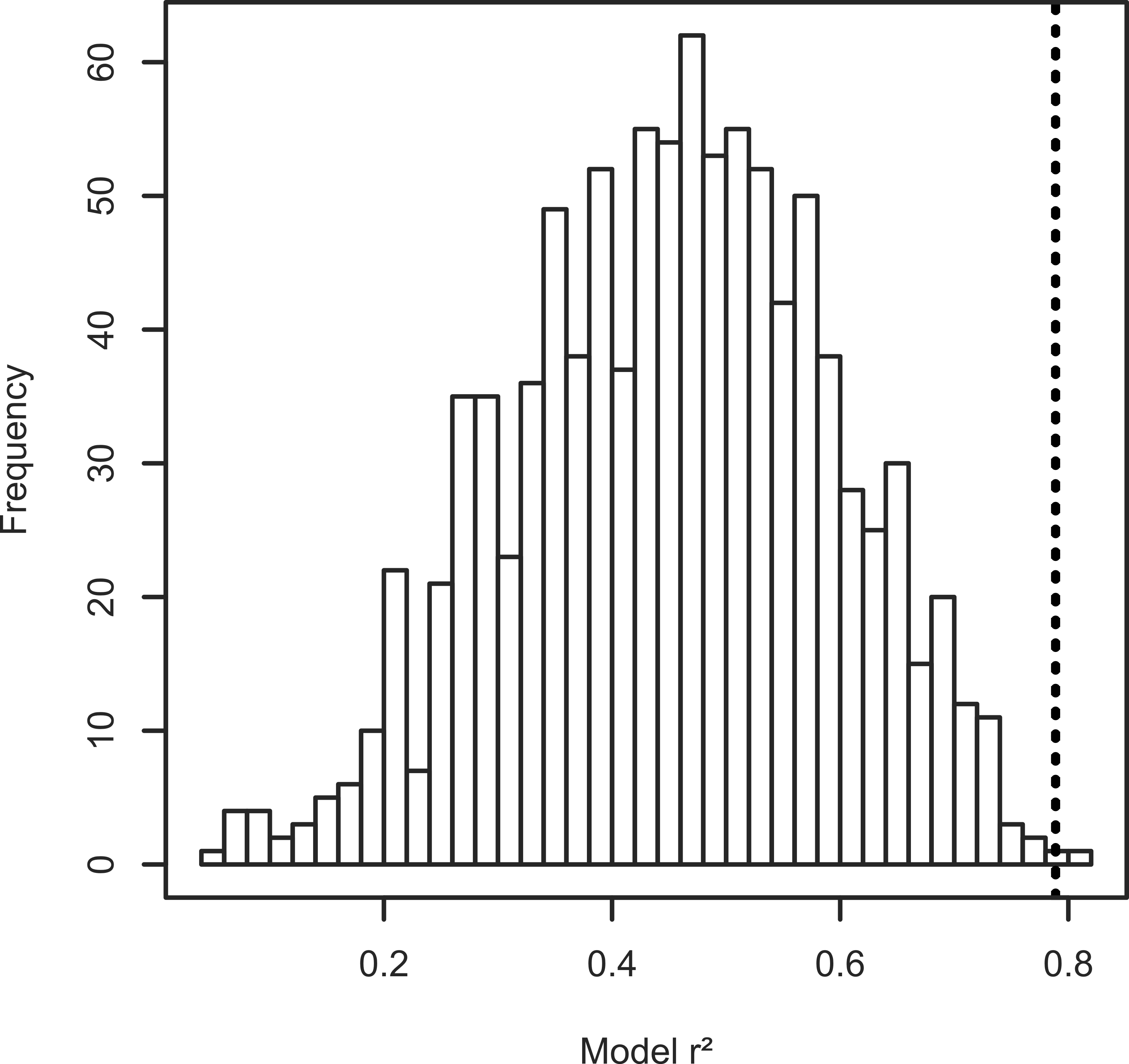

Supplement: Figure S1 — Performance of 1000 transfer functions with simulated environmental variables based on re-mapping of the TP dataset. 998 simulated transfer functions had poorer performance than the actual model r2 based on the observed-inferred TP relationship, as indicated by the dotted line. (TIF) [file pone.0104705.s001.tif]

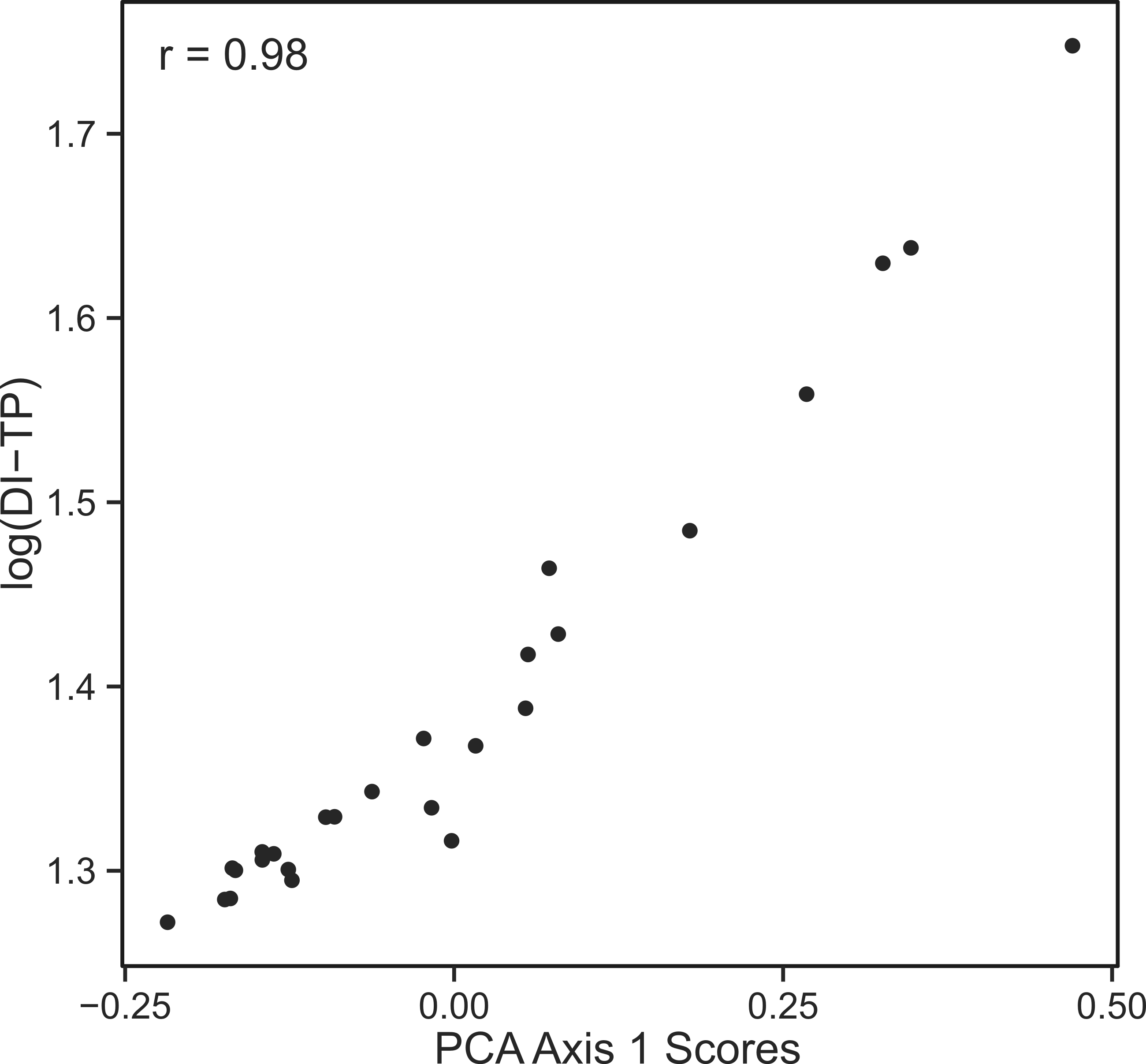

Supplement: Figure S2 — Relationship between Lake Superior downcore DI-TP and PCA axis 1 for diatom data. (TIF) [file pone.0104705.s002.tif]
